# Innovative tools and methods for toxicity testing within PARC work package 5 on hazard assessment

**DOI:** 10.3389/ftox.2023.1216369

**Published:** 2023-07-19

**Authors:** Thalia De Castelbajac, Kiara Aiello, Celia Garcia Arenas, Terje Svingen, Louise Ramhøj, Daniel Zalko, Robert Barouki, Tamara Vanhaecke, Vera Rogiers, Marc Audebert, Michael Oelgeschlaeger, Albert Braeuning, Etienne Blanc, Tamara Tal, Joëlle Rüegg, Ellen Fritsche, Philip Marx-Stoelting, Gilles Rivière

**Affiliations:** ^1^ French Agency for Food Environmental and Occupational Health and Safety, Maisons-Alfort, France; ^2^ German Federal Institute for Risk Assessment, Berlin, Germany; ^3^ National Food Institute, Technical University of Denmark, Kgs Lyngby, Denmark; ^4^ INRAE, Toxalim Research Centre in Food Toxicology, Université de Toulouse, Toulouse, France; ^5^ Université Paris Cité, Inserm unit 1124, Paris, France; ^6^ Department In Vitro Toxicology and Dermato-Cosmetology (IVTD) at the Faculty of Medicine and Pharmacy of the Vrije Universiteit Brussel (VUB), Brussels, Belgium; ^7^ Helmholtz Centre for Environmental Research, Leipzig, Germany; ^8^ Department of Organismal Biology, Uppsala University, Uppsala, Sweden; ^9^ Leibniz Research Institute of Environmental Medicine, Düsseldorf, Germany

**Keywords:** PARC, NGRA, NAMs, hazard assessment, human health

## Abstract

New approach methodologies (NAMs) have the potential to become a major component of regulatory risk assessment, however, their actual implementation is challenging. The European Partnership for the Assessment of Risks from Chemicals (PARC) was designed to address many of the challenges that exist for the development and implementation of NAMs in modern chemical risk assessment. PARC’s proximity to national and European regulatory agencies is envisioned to ensure that all the research and innovation projects that are initiated within PARC agree with actual regulatory needs. One of the main aims of PARC is to develop innovative methodologies that will directly aid chemical hazard identification, risk assessment, and regulation/policy. This will facilitate the development of NAMs for use in risk assessment, as well as the transition from an endpoint-based animal testing strategy to a more mechanistic-based NAMs testing strategy, as foreseen by the Tox21 and the EU Chemical’s Strategy for Sustainability. This work falls under work package 5 (WP5) of the PARC initiative. There are three different tasks within WP5, and this paper is a general overview of the five main projects in the Task 5.2 ‘*Innovative Tools and methods for Toxicity Testing,*’ with a focus on Human Health. This task will bridge essential regulatory data gaps pertaining to the assessment of toxicological prioritized endpoints such as non-genotoxic carcinogenicity, immunotoxicity, endocrine disruption (mainly thyroid), metabolic disruption, and (developmental and adult) neurotoxicity, thereby leveraging OECD’s and PARC’s AOP frameworks. This is intended to provide regulatory risk assessors and industry stakeholders with relevant, affordable and reliable assessment tools that will ultimately contribute to the application of next-generation risk assessment (NGRA) in Europe and worldwide.

## 1 Introduction

The current chemical risk assessment system is challenged by increasingly complex regulatory needs. Emerging challenges include an ever-increasing number of chemicals requiring safety assessment, changes in materials and types of chemicals being produced, as well as complex health effects and aggregate/mixture exposure. These issues, alongside growing ethical concerns related to animal testing, have prompted a shift within chemical risk assessment towards a more mechanism-based predictive paradigm. In this context, Next-Generation Risk Assessment (NGRA) (“the concept of using data from New Approach Methodologies (NAMs) for chemical risk assessment,” Marx-Stoelting et al, 2022) is seen as a promising alternative to conventional risk assessment although implementing innovating hazard and exposure assessment approaches in accordance with regulatory needs has proven to be challenging. The European Partnership for the Assessment of Risks from Chemicals (PARC) has been established specifically to address many of these difficulties. A central idea behind this partnership is that a combined effort from risk assessors, authorities and the scientific community will make real headway towards implementing much needed innovations in testing and assessment for regulatory purposes. The PARC vision is to advance research and share knowledge within the broad field of chemical risk assessment and, by so doing, support the European Union´s Chemicals Strategy for Sustainability and “zero pollution” ambition of the European Green Deal (COM/2019/640 final) (Marx-Stoelting et al, 2022).

The overarching objective of PARC is to consolidate and strengthen the EU´s Research and Innovation (R&I) capacity for chemical risk assessment. This includes safeguarding both human and environmental health. Being a project of significant size, including 200 partner institutions across 28 countries, PARC is structured around nine work packages (WPs). Of these, four are scientific WPs (WP4—Monitoring and exposure, WP5—Hazard assessment, WP6—Innovation in regulatory risk assessment, WP8—Concepts and toolboxes) and five support WPs (WP1—Partnership management and coordination, WP2—A common science-policy agenda, WP3—synergies, collaborations and awareness, WP7—FAIR data, WP9—Building infrastructural and human capacities). The four scientific WPs are interconnected to ensure combined efforts to reach the following three specific objectives (SOs):

SO1—EU, national risk assessors and regulatory entities come together with the scientific community in a cross-disciplinary network to set priorities for R&I in chemical risk assessment;

SO2—European and national risk assessment entities and their scientific networks carry out a joint research and innovation program to respond to the agreed priorities in chemical risk assessment; and.

SO3—European risk assessors, their scientific network and the wider stakeholder community have access to the research and innovation capacities required to implement innovative chemical risk assessment.

**FIGURE 1:**
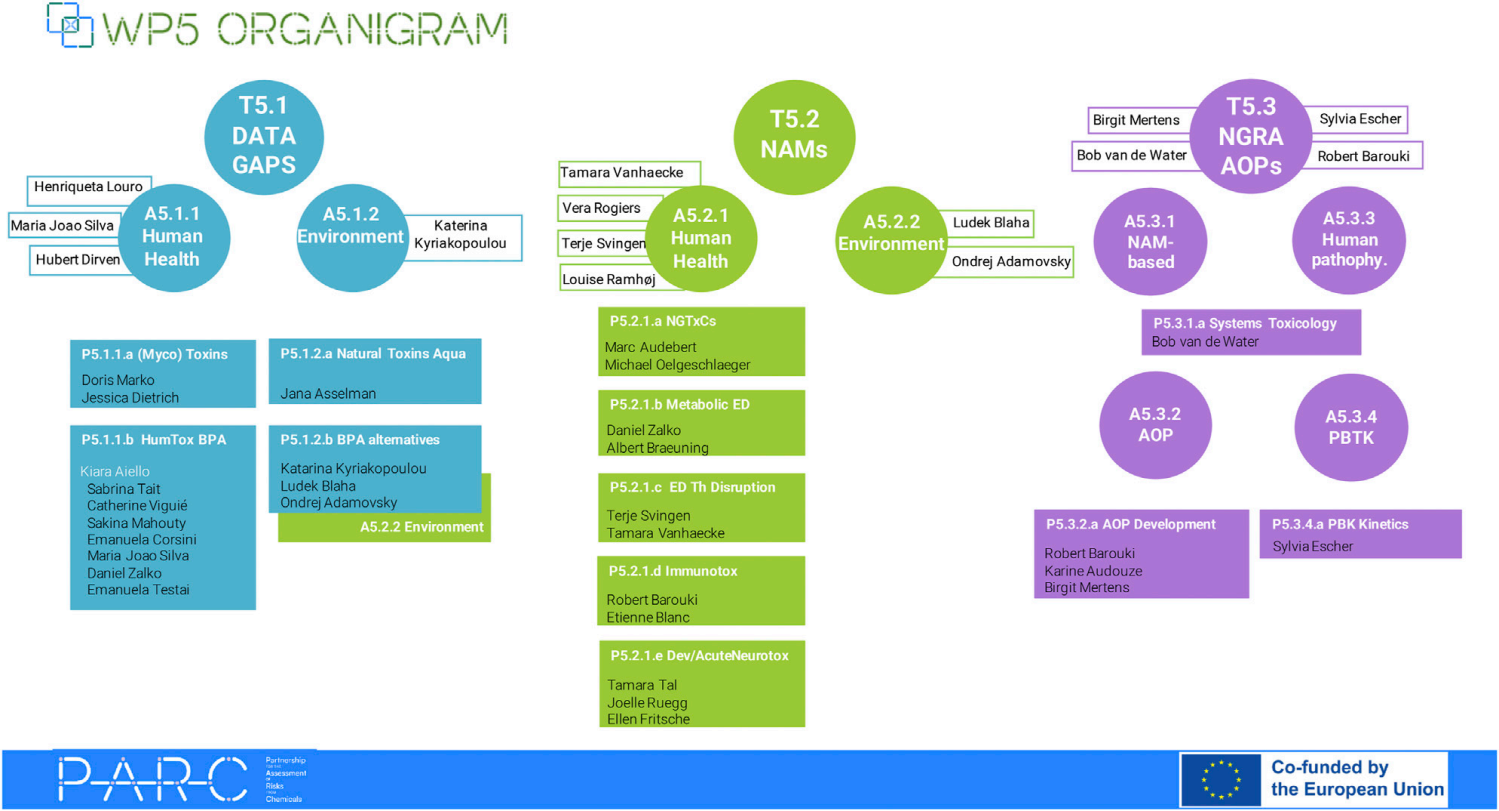
Work Package 5 (WP5) organigram. WP5 is divided into three Tasks (T): T5.1 Closing data gaps of concern (Data gaps in brief); T5.2 Innovative tools and methods for toxicity testing (NAMs in brief); T5.3 Quantitative systems toxicology and AOP development. In each Task there are two Activities, and in each activity different projects. The task/activity described in this document is T5.2/A5.2.1 and its primary focus is on human health, with environmental health being covered by Task 5.2.2.

Within PARC, WP5 will develop NAMs for hazard assessment, provide data to fill gaps in knowledge on poorly characterized contaminants or new emerging hazards, and promote the use of innovative methods and tools to contribute to the integration of new technologies. WP5 is divided into three overarching tasks and 12 projects (Figure 1). One of these tasks is Task 5.2 ‘*Innovative Tools and methods for Toxicity Testing’*, which aims to improve the current hazard characterization by establishing comprehensive testing strategies that logically combine novel methods with well-established approaches, preferably in a tiered manner. Task 5.2 is closely linked to the task ‘Quantitative systems toxicology and development of new AOPs’, which addresses the “physiology-toxicology crosstalk” (Heindel et al, 2017). In the following, we provide an overview of the activities under this Task 5.2, with a focus on Human Health, to promote open science and engage the broader scientific community to foster increased collaborations. Each project will be described in detail in separate papers in the same special issue of Frontiers https://www.frontiersin.org/research-topics/48691/european-partnership-on-the-assessment-of-risks-from-chemicals-parc-focus-on-new-approach-methodologies-nams-in-risk-assessment#articles.

Activities under this task of WP5 will provide innovative methods (individual assays) and methodologies (such as IATAs) and thereby directly aid a wide range of EU regulations, such as REACH (EC) No 1907/2006, the Food Contact Materials regulation (EC) No 1935/2004, the Plant Protection Products regulation (EC) No 1107/2009b, the Cosmetics regulation (EC) No 1223/2009, the Classification, labelling and packaging (CLP) regulation (EC) No 1272/2008, the Biocides regulation (EC) No 528/2012, the EU chemicals strategy for sustainability (COM/2020/667 final), and the European Green Deal (COM/2019/640 final). The stated aims will be achieved by evaluating the relevance of a suit of technologies ranging from genomics, transcriptomics, and proteomics, to state-of-the-art *in vitro* assays and human stem cell technologies, with an initial focus on five prioritized endpoints.

1. Non-genotoxic carcinogenicity;
2. Metabolic endocrine disruption;
3. Endocrine disruption (thyroid);
4. Immunotoxicity; and
5. (developmental and adult) neurotoxicity.

Additionally, tools and methods will be developed to identify toxicity drivers in mixtures and to support the grouping of chemicals, including the application of read-across. Predictive *in vitro* and *in silico* methods will be developed and refined, to ultimately be used for identifying and characterizing specific hazards. This will facilitate one of the overall objectives of the PARC initiative—to develop tools and strategies in accordance with regulatory needs—by establishing comprehensive testing strategies based on new and improved NAMs for human-relevant predictive toxicology. A clear vision is to increase overall scientific and regulatory confidence in NAMs.

## 2 Non genotoxic carcinogens

### 2.1 Context

Some substances may induce cancer without being detectable in regulatory genotoxicity assays, for instance by acting as tumor promoters, modulators of nuclear receptors or as inducers of tumor progression and metastasis or tissue-specific toxicity, as well as immune and inflammatory responses. Currently, the assessment of a potential hazard from chemical exposure that might lead to cancer relies on long-term rodent-based bioassays, which are carried out mainly in accordance with OECD TG451 and TG453. However, these standards require a great number of animals and their human relevance has been questioned (Elcombe et al, 2014; Felter et al, 2018; Holsapple et al, 2006). There is therefore an urgent need for a transition to *in vitro* and more human-relevant approaches to exploit our current understanding of cancer and use the latest non-animal methods available in a modern safety assessment toolbox. The OECD and other international regulatory authorities have acknowledged the lack of such methods in carcinogenicity testing (Jacobs et al, 2020).

### 2.2 How?

Human relevant-NAMs (transcriptomic approaches, high-content analysis cell painting, among others) and *in silico* tools will be used and optimized to investigate a range of systems (liver, breast, colon, adipocytes) and develop a non genotoxic carcinogens (NGTxCs) integrated approaches to testing and assessment (IATAs). Physiological and toxicological relevant information pertaining to the adversity of potential carcinogenic effects will be integrated using increasingly complex methods (2D cell culture up to 3D spheroids and zebrafish as a simple vertebrate model system).

### 2.3 Innovation and regulatory impact

The added value of this project will be significant. From a risk assessor’s perspective this project will, for the first time, allow for a rapid mechanistic NGTxCs hazard identification of a high number of substances, as well as the screening of mixtures.

## 3 Metabolic endocrine disruption

### 3.1 Context

There are over 50 million people in Europe suffering from metabolic disorders, and latest estimates from the WHO for European Union countries indicate that 30%–70% of adults are overweight and 10%–30% obese (World Health Organization, 2020). Recent research into endocrine disrupting chemicals (EDCs) has demonstrated that several EDC, referred to as “metabolism disrupting chemicals” (MDC) can induce a lasting disruption of endogenous metabolism. MDCs are suspected of being linked to obesity and related metabolic disorders such as type II diabetes and non-alcoholic fatty liver disease (Lind et al, 2016). Despite this potential major role in the worldwide epidemics of metabolic disorders, there are currently no specific regulatory *in vivo* or *in vitro* tests allowing to identify their adverse effects. The need for testing developments in the field of metabolic disorders has been highlighted in recent work by EURION cluster projects (Audouze et al, 2020; Küblbeck et al, 2020; Legler et al, 2020).

### 3.2 How?

A comprehensive exploration of nuclear receptor-driven effects will be carried out using stably transfected cell lines, expanding on the on-going pre-validation of transient cell lines already in Horizon-2020 EURION cluster of projects. Specific compounds will be selected from the PARC priority list (BPA alternatives), the EURION cluster, or data gaps identified by EU agencies such as ECHA and EFSA. The assessed substances include major metabolites and breakdown molecules, allowing further MoA exploration, as well as adverse outcome pathway (AOP) and read-across development. NAMs encompassing hepatic and non-hepatic *in vitro* systems, including multi-tissue cross-talk, will be developed, opening for a refined assessment of the obesogenic effects of MDC. Ultimately, this project will bridge major data gaps in the field of metabolic disruption through the development of accurate NAMs based on human as well as non-human models.

### 3.3 Innovation and regulatory impact

New methods will be developed for identifying MDCs with outcomes expected to be of great use for risk assessors as there are currently no specific regulatory *in vivo* or *in vitro* tests allowing to identify their adverse effects, and the role of environmental stressors in metabolic disorders is being increasingly recognized. The regulatory robustness of some promising methods developed in EURION projects will be increased, and research on key-pathways involved in metabolic disruption will be conducted.

## 4 Endocrine disruptors—Thyroid hormone system disruption

### 4.1 Context

Despite recent regulatory advances such as the revised OECD guidance document 150 adopted by ECHA, current testing strategies and regulations are widely regarded as inadequate when it comes to identifying thyroid hormone system disruptors (THSDs) (Courderq et al, 2020; Gilbert et al, 2020; Kortenkamp et al, 2020; Noyes et al, 2019). Although some *in vitro* assays (non-OECD TGs) for certain molecular initiation events relevant for THSD are available, and are currently in the process of regulatory validation within the OECD, there remains a pressing need to develop more extensive test batteries of NAMs that cover a broader range of mechanisms linking exposure with adverse outcomes in humans and animals.

### 4.2 How?

To facilitate the development of thyroid hormone system specific NAMs, we will use a variety of approaches. State-of-the art multi-organ RNA-sequencing (toxicogenomics) will be used to characterize *in vivo* mechanisms of action for targeted NAMs development. Human-relevant *in vitro* test systems will be characterized and developed for key events of relevant toxicological pathways, including elaboration of species-specific differences by combining human-derived *in vitro* assays, *in silico*, zebrafish and rats as well as an effort to leverage data from non-mammalian vertebrates to inform on hazards for human health.

### 4.3 Innovation and regulatory impact

The project will deliver valuable contributions to chemical safety assessors by improving and developing NAMs for detection of THSDs. By filling critical knowledge gaps on the effects and mechanisms of THSDs in developing organisms, as well as interpretations in the AOP framework, the aim is to enable an improved assessment of THSD properties. This will contribute to the detection of hazardous substances with focus on EDCs and THSDs, thus adhering to the zero-pollution ambition of the European Green Deal (COM/2019/640 final), as well as the Chemicals Strategy for Sustainability (COM/2020/667 final) set out by the European Commission.

## 5 Immunotoxicity

### 5.1 Context

Immune dysregulation ranges from acute uncontrolled inflammation, chronic inflammation, allergic to sensitization reactions, autoimmunity, and immunosuppression. Since exposure to certain chemicals can cause such dysregulations, it is critical to develop tools and test that distinguish between normal adaptive conditions and immune dysregulations, for regulatory purposes. As such, this project aims to characterize NAMs addressing three major endpoints in immunotoxicity: organ-related immunotoxicity with a focus on respiratory toxicity and sensitization; immunosuppression, in particular through response to vaccination; characterizing new models for cellular immunotoxicity assessing key events. The major outcome will be the development of NAMs in these immunotoxicity areas.

### 5.2 How?

This project will deliver a set of *in vitro* systems to investigate key events in the immune system function or dysfunction. New parameters will be developed to better explore the immune system cellular content and activity, cytokine and antibody production and release, as well as the effect of signaling. Different primary cell systems and cell lines as well as epithelial and immune cell co-cultures will be characterized. Reference chemicals will be used and once the NAMs are developed, PARC priority substances will be tested when relevant.

### 5.3 Innovation and regulatory impact

This project will aid risk assessors by providing innovative methods to allow for rapid mechanistic hazard identification of a high number of substances, contributing to the detection of hazardous substances. Additionally, the methods will enable the screening of mixtures, and thereby supporting the many EU strategies referred to in the Introduction.

## 6 Neurotoxicity

### 6.1 Context

Developmental (DNT) and Adult (ANT) Neurotoxicity guideline studies are extraordinarily resource-intensive and therefore not suited for studying the adverse effects of a large number of chemicals. In REACH, DNT guideline studies are not mandatory and ANT testing is not performed for low-tonnage compounds. There is international consensus, strongly supported by EFSA and the OECD (EFSA PPR Panel et al, 2021), that DNT testing must be made faster and more human-relevant by implementing an alternative DNT testing battery for regulatory purposes. The current state-of-the-art is the use of a DNT *in vitro* test battery (DNT IVB) comprised of several human- and rat-based *in vitro* test methods covering key neurodevelopmental processes which include neural progenitor differentiation, proliferation, neurite outgrowth in CNS neurons and neural crest cells, neural crest cell migration, and oligodendrocyte differentiation (Masjosthusmann et al, 2020). While this represents a step forward towards establishing an alternative DNT testing regime, there are significant gaps in the DNT IVB including human synaptogenesis, human neural network formation, myelination, and correlates for the rodent *in vivo* studies including endpoints that measure behavior, sensory function, learning, and memory.

### 6.2 How?

The key strategy is to provide a comprehensive battery of NAMs that fills the gaps in the current DNT *in vitro* battery (IVB) and, for ANT, to set up such a battery. In both cases, NAMs will be added that cover missing endpoints and key DNT/ANT modes-of-action (e.g., endocrine disruption, epigenomic alterations). For *in vitro* NAM development, the focus lies solely on human cells which circumvent the problem of species specificity. In addition, zebrafish embryos are used as an alternative, whole organism, low-cost screening tool with integrated nervous system functions capable of detecting chemical effects on missing, complex endpoints including behavior, sensory function, learning, and memory. As there are few neurotoxicity adverse outcome pathways (AOP) available, new AOPs will be generated. Newly established or optimized methods in this sub-project will undergo test method set-up and mechanistic validation.

### 6.3 Innovation and regulatory impact

Additional IVB NAMs developed for DNT/ANT will provide risk assessors with rapid mechanistic hazard analysis. This will contribute to, along with mixture screening in these NAMS, the European Green Deal (COM/2019/640 final), as well as the Chemicals Strategy for Sustainability (COM/2020/667 final).

## 7 Concluding remarks

This paper gives a brief overview of Task 5.2 of PARC, named Innovative Tools and Methods for Toxicity Testing. Research and innovation towards NAMs and, more broadly, NGRA, is one of the major objectives of the PARC initiative. It aims to review current approaches and provide novel practices and methods, foster NAMs regulatory uptake, all whilst following the 3 R (Reduction, Refinement and Replacement) principles. With its many activities and interactions across Tasks and WPs, it is beyond the scope of this short report to outline all the many facets of the project; rather, the focus is on providing a brief overview of the Task’s mandate and activities to promote the underlying mission of PARC, which is to engage a broad range of professionals from various fields to develop innovative solutions to modern chemical testing strategies in an open, collaborative manner. We have highlighted the five main endpoints that are in focus for the initial few years of the 7-year project duration. The vision is to complete some of these activities within 3–4 years as well as include additional endpoints as the project progresses. For these updates and additional information on the PARC project, we would like to refer to the open website, https://www.eu-parc.eu/.
